# Topical Turmeric Microemulgel in the Management of Plaque Psoriasis; A Clinical Evaluation

**Published:** 2015

**Authors:** Golnaz Sarafian, Minoo Afshar, Parvin Mansouri, Jinous Asgarpanah, Kosar Raoufinejad, Mehdi Rajabi

**Affiliations:** a *Department of Clinical Pharmacy, Pharmaceutical Sciences Branch, Islamic Azad University, Tehran, Iran. *; b *Department of Pharmaceutics, Islamic Azad University Pharmaceutical Sciences Branch, Tehran, Iran.*; c *Skin and Stem Cell Research center, Tehran University of Medical Sciences.*; d *Department of Pharmacognosy, Islamic Azad University Pharmaceutical Sciences Branch, Tehran, Iran.*

**Keywords:** *Curcuma longa *L*.*, Plaque psoriasis, Topical turmeric microemulgel, Dermatology Life Quality Index (DLQI) Questionnaire, Psoriasis Area &amp; Severity Index (PASI) score

## Abstract

Psoriasis is an autoimmune and recurrent chronic inflammatory skin disease. About 1-3% of the world wide populations are affected. The characteristic features are hyperprolifration of keratinocytes leading to redness, thickening and scaling of epidermis followed with itching and appearance of the lesions which in most cases bother the patients medically and psychologically. Psoriasis is symptomatically treated by the range of oral and topical medications, however, major side effects in some cases are associated with them. Based on several studies,* Curcuma longa *can inhibit several inflammatory enzymes mainly involved in the inflammatory process of Psoriasis. Therefore, we decided to target this well-known herbal agent with fantastic safety profile to be formulated as a novel topical microemulgel.

The clinical and therapeutic benefit of this novel topical formulation was evaluated on 34 patients with mild to moderate plaque psoriasis in a randomized, prospective intra-individual, right–left comparative, placebo-controlled, double-blind clinical trial. The Dermatology Life Quality Index (DLQI) Questionnaire and Psoriasis area & severity index (PASI) score as well as photos before and after treatment was used to evaluate the outcomes. The results show that the clinical and quality of life parameters in treated lesions in comparison with untreated lesions have improved (P<0.05). The reported side effects were also recorded and were trivial. Based on our findings, the proposed microemulgel may well be considered as an alternative in some patients and most likely as an add-on therapeutic option for many patients suffering with plaque psoriasis.

## Introduction

Plaque psoriasis is a hyper proliferative, non infectious, inflammatory skin condition characterized by recurrent exacerbations and remissions of thick end, erythematous, and scaling lesions (1). Psoriasis sufferers are at risk of other serious comorbidities and therefore complicates the management of this condition and in some cases increase the risk of early death. Cardiovascular disease (CVD), crohn’s disease, chronic obstructive pulmonary disease (COPD), lymphoma, depression and metabolic syndrome are reported more commonly in psoriasis patients (2). The national psoriasis foundation reported that 33% of patients with mild disease and 60% of patients with moderate-to-severe psoriasis reported that their disease was a significant problem in their everyday life (2). Psoriasis can be effectively controlled by various medications, either used alone or in combination. About 75% of patients with mild-to-moderate psoriasis are responsive to topical treatments (3). However, a variety of conventional therapies have generated limited efficacy with frequent side effects. Other alternative therapies for psoriasis are; light therapy, nutritional therapy, hydrotherapy, detoxification, fasting, and colon therapy. Ayurvedic medicine (Ayurveda) is the most widely accepted alternative medicine for psoriasis. Medicinal plants, herbs, spices and herbal remedies are known to ayurveda for long time (4, 25).

Turmeric (*Curcuma longa*) is extensively used as a spice, food, preservative and traditional medicine and as a household remedy for various diseases in Asian countries, including China and South East Asia.

 Curcumin, a yellow pigment from *Curcuma longa* is a major component of turmeric with immune-modulating, anti-inflammatory, wound-healing, antibacterial, antitumour, anti-carcinogenic, and antioxidant properties (5, 6, 21, 24). This agent has shown some significant effect on psoriasis with properties related on numerous receptors to which curcumin binds. These include 5-LOX, xanthine oxidase, thioredoxin reductase, COX-2, *p*-glycoprotein, GST, PKA, PKC, cPK, PK, Ca²^+^- dependent protein kinase (CDPK), and glutathione (21). Furthermore, curcumin induced suppression of phosphorylase kinase activity which correlates with the resolution of human psoriasis (7).

As a novel approach, in our investigation, the topical microemulgel was considered as a great potential product for an effective and safe way to administer turmeric for the treatment of skin diseases such as psoriasis.

Patel *et al*. 2009 has investigated the effect of curcumin gel in rats for its anti-inflammatory effects. The penetration percutaneous flux of curcumin through the rat epidermis was enhanced by the used of menthol in the formulation (8).

In more recent study by khiljee *et al*. (2010) the release of topical turmeric gel, ointment and micro emulsion through the franz cells were evaluated and compared against each other. The turmeric micro emulsion had the fastest release through nylon membrane and cellulose membrane compared with the gel or ointment (9).

The purpose of this double-blind, placebo controlled study was to evaluate the clinical efficacy of turmeric extract with fantastic safety profile in a novel topical microemulgel in patients with plaque psoriasis.

## Experimental

*Patients and methods*

Initially, forty patients (this number of patients was based on similar studies investigating other herbal topical preparations) entered in to the study with the male to female ratio of 14 to 20 respectively. The number of patient was based on similar studies when evaluating the clinical efficacy of novel topical herbal remedies in human subjects. All patients were between the ages of 18-60 years with the mean of 31.7 years old (Table 1). Patients were diagnosed with mild to moderate bilateral symmetrical lesions of stable plaque psoriasis on legs and arms by the dermatologist. The inclusion criteria were; stable plaque psoriasis diagnosed clinically by a consultant dermatologist as symmetrically distributed psoriatic plaques which had been stable in extent and severity for at least 2 months on systemic therapy and had not used topical therapy for the past 2 weeks. Patients who had been treated aggressively by beta blockers or those patients with other complications such as lymphoma and infections were excluded. Additionally, this study was only approved by the ethics committee based on extension of exclusion criteria to exclude the breast feeding or expecting mothers**. **This was a randomized, prospective intra-individual, right–left comparative, placebo-controlled, double- blind pilot study lasted 9 weeks in total for each individual patient. Patients were advised to apply the turmeric microemulgel twice daily versus placebo which was consist of vehicle alone. Patients were advised to apply a thin layer of drug or placebo on entire surface of the selected lesion (selected lesions could be in different size of distribution). The trial was registered in Iranian Registry of Clinical Trials (IRCT registration number: IRCT201304203106N13). The study was approved by Azad University of Pharmaceutical Sciences Ethics Committee (No: 13993). Informed consents were obtained from each patient before they were included in our study.

Hydro alcoholic turmeric extract was the main active ingredient. The extraction carried out by the percolation method as disclosed by the SOHA JISSA Co (Salmanshahr, Mazandaran, Iran).

Both active and placebo preparations were made identical in their presentation and supplied in a 50 g tubes with storage condition at ambient temperature. Patients were given 50 g tubes (active/placebo) for a maximum of 3 weeks supply at a time, clearly labeled for left or right lesions. Patients were instructed to use them twice daily. In order to asses and compare the clinical efficacy, the psoriasis area and severity index (PASI) score, photography and quality of life questionnaire known as Dermatology Life Quality Index (DLQI) were used. The level of adherence and basic follow ups with treatments were carried out on weekly basis by phone and in person. Additionally, all patients had 24/7 access helpline number to contact during the trial and were examined physically in the multi disciplinary dermatology clinic every 3 weeks for the period of 9 weeks. SPSS T-test, chi-squared test were used for comparing quantitative values. 

*Preparation and evaluation of microemulgel*

Hydroalcoholic turmeric extract was the main active ingredient. The extraction carried out by the percolation method as disclosed by the SOHA JISSA Co.

The pharmacological activities of turmeric have been attributed to its hydroalcoholic extract, which contain curcuminoids. A variety of methods have been reported for the quantification of curcumin. HPLC coupled with UV detection is the most common method for determination of curcumin in turmeric samples. In the present study, a sensitive HPLC–UV method was established for the qualitative and quantitative analysis of curcumin in hydroalcoholic extract of turmeric.

Curcumin amount in raw and final products was calculated using the previously validated and published method (22). Briefly, separation was carried out on a Shimadzu HPLC system (Shimadzu, Kyoto, Japan), which was equipped with an SCL-10AVP system controller, LC-10 ADVP pump, DGU-14A degasser, and a SPD-M10AVP PDA detector. The peak areas were integrated automatically by computer using a Shimadzu Class VP software program. A 20 µL volume of sample was introduced into a Rheodyne model 7725i injector. The elution was performed on a C_18_ column (150 mm×4.6 mm, 5 µm particle size) from Teknokroma (Barcelona, Spain). All analyses were carried out at the column temperature of 30±1^◦^C under isocratic conditions with a mobile phase of acetonitrile: acetic acid solution (pH= 3) (30:70, v/v), and a flow-rate of 2.0 mL/min, using PDA detection at 240 nm. All solvents and reagents were of gradient and analytical grade respectively and were purchased from Merck (Darmstadt, Germany). HPLC grade water was obtained through a Milli-Q system (Millipore, Milford, MA, USA) and was used to prepare all the solutions. The pharmaceutical dosage form (microemulgel 0.5%) were prepared based on our previously patented formulation (IR-80034).

## Results

34 patients from initially recruited 40 patients completed the study. The rate of compliance was reasonable probably due to excellent and robust follow up system and due to the tolerability to the formulated topical preparation. All subjects completed the 9 weeks active treatment, and during that period progressive reduction of thickness, followed by decrease erythema, pruritus, resulting in moderate to acceptable improvement and in some cases significant resolution of psoriatic lesions. The mean duration of disease prior to participation in the clinical trial was 11.5 years. This is the duration where patients were clinically diagnosed as suffering with psoriasis by the consultant dermatologists. 58.8% of patients were diagnosed before the age of 30 years old and 41.2% were diagnosed after the age of 30 (Table 1). 

**Table 1:** Characteristics of the patients in our sample selection and followed for 9 months

**Sex**	**Frequency **	**%Frequency**
Male Female	20 14	58.8 41.2
Onset of disease (years)		
Before the age of 30 After the age of 30	20 14	58.8 41.2
Family history of psoriasis		
Yes No	18 16	53 47

**Figure 1:**
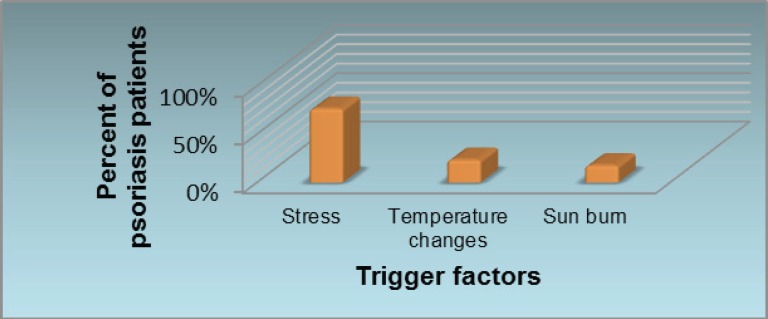
Most common causes of exacerbation of psoriasis as reported by patients are stress, temperature changes and sun burn.

Initially using psoriasis questionnaire the causes and trigger factors were evaluated. Stress, sun burn and temperature changes are reported to be the most common trigger factors for the exacerbation or worsening of psoriasis (Figure 1). 

**Figure 2:**
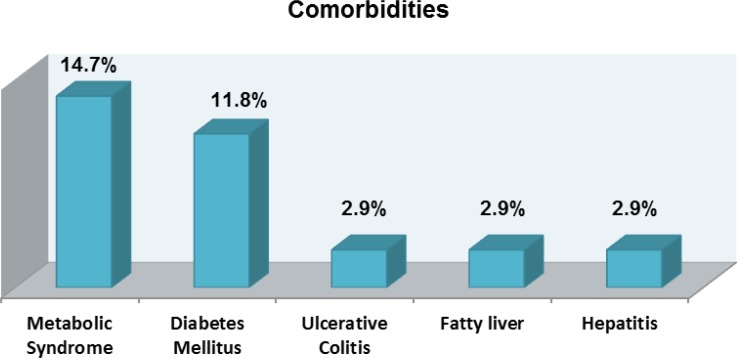
Most common comorbidities reported by psoriatic patients

Psoriasis has been traditionally considered to be a disease of skin, however, it is now thought to be the cause for systemic inflammation and linked to be the source for other comorbidities such as cardiovascular disease, liver, respiratory and hematological complications. In particular the most common complications were metabolic syndrome and diabetes mellitus with 14.7% and 11.8% respectively. In lower number of 2.9% other comorbidities such as ulcerative colitis, fatty liver and hepatitis are reported (Figure 2). 

**Figure 3:**
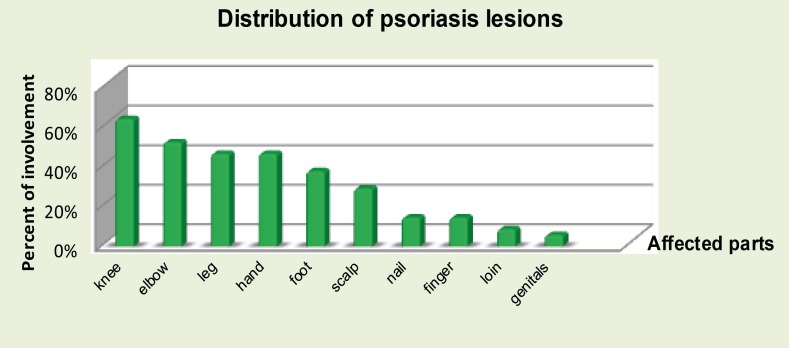
Pattern for distribution of psoriasis in individual patients

In our sample size, the distribution of psoriasis lesions on individuals were recorded at the baseline. Majority of body parts which are affected by this condition are knees and elbows and that corresponds to the findings by various literatures and text books as the most common reported sites to be affected (Figure 3).

**Table 2:** Quality of life parameters in plaque psoriasis patients on week 0 and 9 using Dermatology Life Quality Index (DLQI) Questionnaire

	** Very much **	** A lot**	** A little**	** Not at all**
Itching, painful/stinging				
Week 0 Week 9	4 (11.8%) 0 (0%)	8 (23.5%) 4 (11.8%)	15 (44.1%) 6 (17.6%)	7 (20.6%) 24 (70.6%)
Embarrassments/self consciousness				
Week 0 Week 9	4 (11.8%) 1 (2.9%)	8 (23.5%) 4 (11.8%)	15 (44.1%) 10 (29.4%)	7 (20.6%) 19 (55.9%)
Interference with daily activities				
Week 0 Week 9	3 (8.8%) 0 (0%)	1 (2.9%) 1 (2.9%)	8 (23.5%) 6 (27.6%)	22 (64.7%) 27 (79.4%)
Impact on clothing				
Week 0 Week 9	8 (23.5) 1 (2.9%)	8 (23.5) 4 (11.8%)	9(26.5%) 11(32.4%)	9 (26.5%) 18 (52.9%)
Influence on Social or leisure activities				
Week 0 Week 9	3 (8.8%) 2 (5.9%)	6 (17.6%) 2 (5.9%)	8 (23.5%) 7 (20.6%)	17 (50%) 23 (67.6%)
Impact on sport Prevention from work/studying				
Week 0 Week 9	1 (2.9%) 1 (2.9%)	4 (11.8%) 2 (5.9%)	3 (8.8%) 5 (14.7%)	26 (76.5%) 26 (76.5%)
Problems with partner/friends/ relatives				
Week 0 Week 9	1 (2.9%) 0 (0%)	6 (17.6%) 4 (11.8%)	13 (38.2%) 12 (35.3%)	14 (41.2%) 18 (52.9%)
Sexual difficulties				
Week 0 Week 9	3 (8.8%) 0 (0%)	2 (5.9%) 2 (5.9%)	2 (5.9%) 3 (8.8%)	27 (79.4%) 29 (85.3%)
Time consuming				
Week 0 Week 9	0 (0%) 2 (5.9%)	5 (14.7%) 4 (11.8%)	12 (35.3%) 8 (23.5%)	17 (50%) 20 (58.8%)

**Figure 4:**
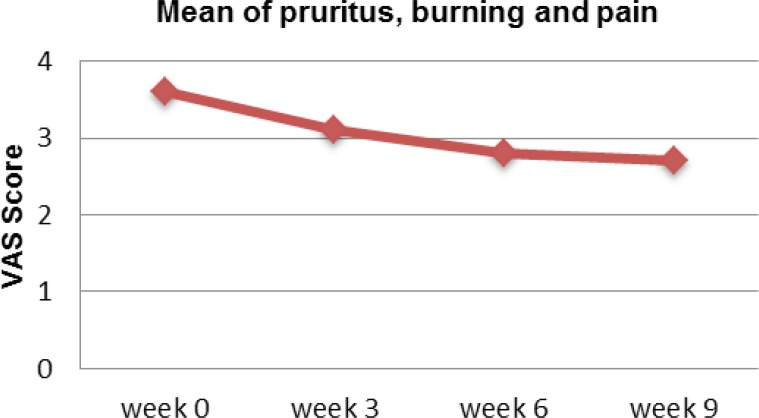
Mean visual analog scale for pruritus, burning and pain on week 0, 3, 6 and 9 using the proposed microemulgel.

Visual Analog Scale (VSA) is used to assess the level of pruritus, burning and pain associated with the psoriasis. Overall, patients experienced reduction of VAS when used the proposed turmeric microemulgel (Figure 4).

PASI is the current gold standard for assessment of the psoriasis. The PASI was used to measure the average redness, thickness, and scaliness of the lesions (0–4 scale for each parameter) and the areas of involvement (0–6 scale). 

**Figure 5:**
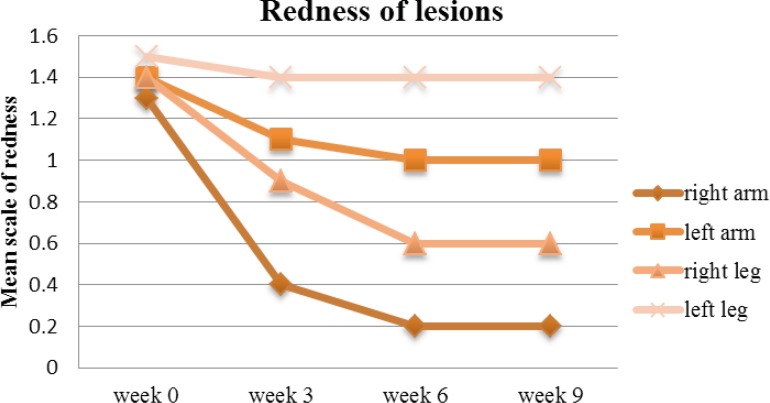
Mean redness of arm and leg lesions treated with drug (right arm & leg) and placebo (left arm & leg) on week 0, 3, 6 and 9.

On week 9, the mean redness score gradually reduced from 1.3 to 0.2 on the right arm (P<0.05), against the left arm where the reduction was not significant enough (from 1.4 to 1), albeit reduction was gradual. Similarly, the redness associated with the lesions on the legs were following the same pattern of reduction and therefore the difference between left and right sides were prominent (Figure 5). 

**Figure 6:**
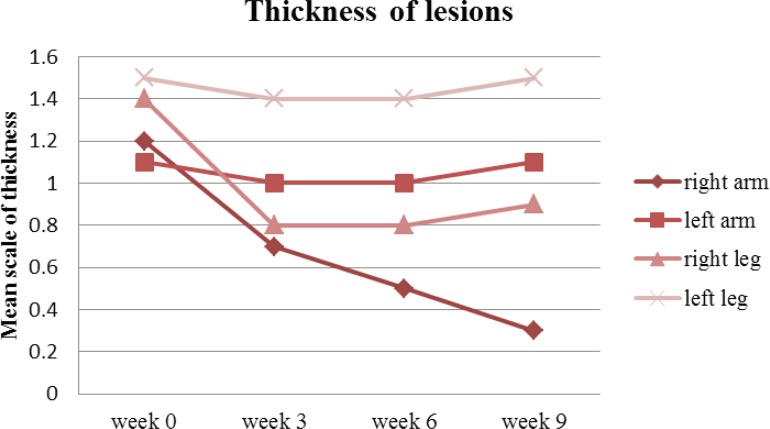
Mean thickness of arm and leg lesions treated with drug (right arm & leg) and placebo (left arm & leg) on week 0, 3, 6 and 9

On week 9, thickness of the lesions reduced from 1.1 to 0.3 on the right arm (P <0.05) against the left arm where it only reduced from 1.2 to 1.1. At the same time, the thickness on the right leg reduced from 1.4 to 0.9 when compared against the left leg which only reduced insignificantly from 1.5 to 1.4 (Figure 6). 

Overall, the right side lesions were improved significantly regardless of the affected part of the body (P<0.05).

**Figure 7:**
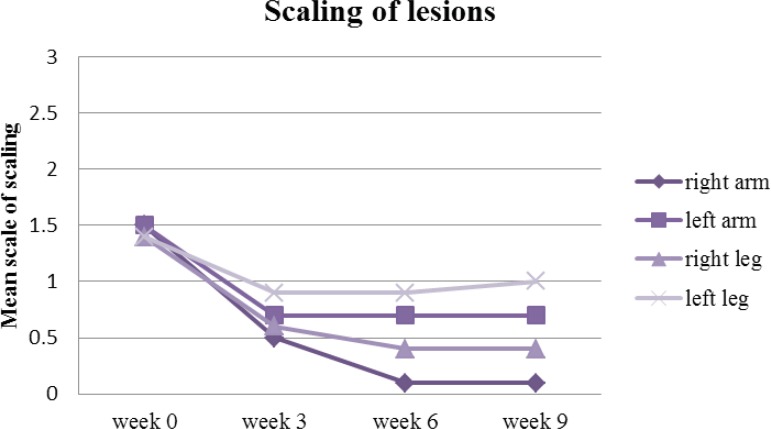
Mean scaling of arm and leg lesions treated with drug (right arm & leg) and placebo (left arm & leg) on week 0, 3, 6 and 9

 The turmeric microemulgel was noticeably more effective than the placebo in decreasing the scaling lesions. From the beginning to the end of study, the scaling of the lesions were reduced from 1.5 to 0.1 on the right arm (P<0.05) against the left arm where it reduced from 1.5 to 0.7 up to week three and then remained roughly the same all the way until the end of the study. The scaling on the right leg reduced from 1.4 to 0.4 (P<0.05) when compared against the left leg which only reduced insignificantly from 1.4 to 1 (Figure 7).

**Figure 8:**
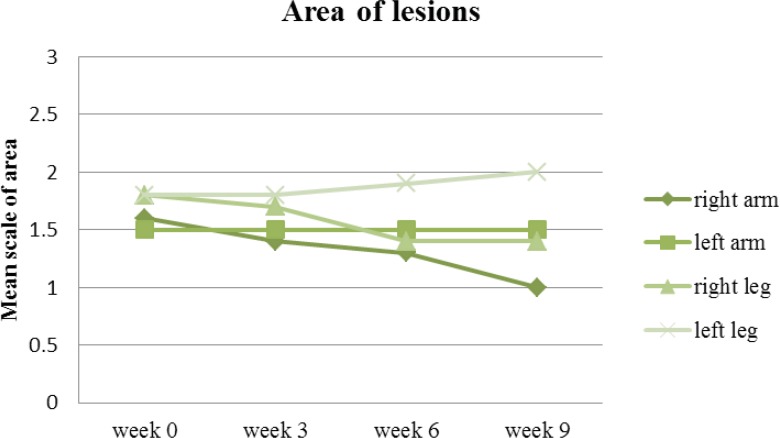
Mean area of arm and leg lesions treated with drug (right arm & leg) and placebo (left arm & leg) on week 0, 3, 6 and 9

The area of lesion is the final parameter in the PASI scoring system. Equally, the area of lesions at the end of the follow up on the right side of the body was meaningfully improved (P<0.05) in comparison to the left side where the area of involvement more or less remained the same as far as the lesions on the arms were concerned. The involved area of lesions on the left leg was even increased (the lesions worsened) in some respect in comparison to the beginning of the study (Figure 8). 

**Figure 9:**
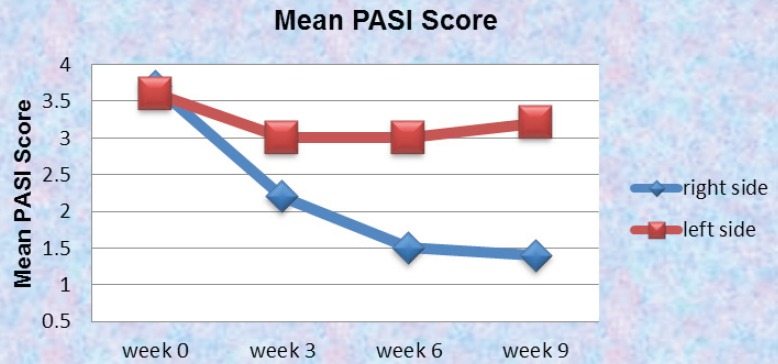
Mean Psoriasis Area and Severity Index (M-PASI) score on week 0, 3, 6 and 9 using 0.5% turmeric microemulgel on the right side against placebo on the left side

Overall, the mean PASI score at baseline between the two sides were 3.6 and 3.7 for the right and left side of the body respectively. That was simply due to the fact that in our patient selection we were extra careful to select those lesions which were identical in their presentations and size (the involved area). In our investigation, the side treated with drug improved in PASI and that level of steady improvement was observed all the way in the clinical trial (P<0.05). On the other hand the side treated with placebo had a mild improvement up to week three and then remained almost the same all the way in to week 9. This could be due to the fact that our placebo was acting as a moisturizer and that influence was sufficient enough to improve the PASI in less significant manner in comparison to the side treated with drug where it had the benefit of the properties of the active ingredient with more meaningful improvement of the PASI score (Figure 9). 

**Figure 10:**
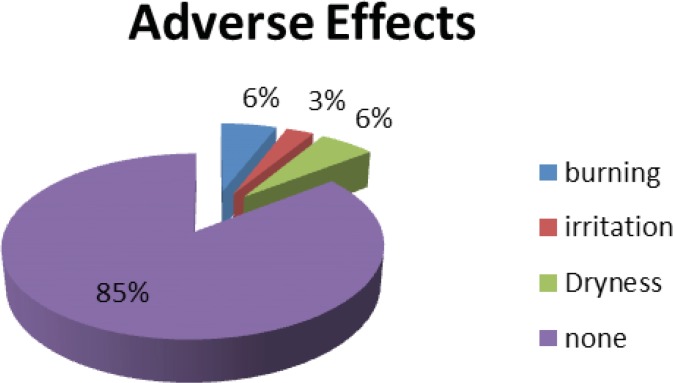
The most common adverse effects reported by patients up to 4 weeks post trial.

As a part of novel clinical trial and based on good clinical trial practice we gathered any adverse effects experienced by the patients thorough out our study and at least for 4 weeks post trial period. All patients had access to 24 hours help line for reporting any undesirable side effects. Based on those reported side effects 85% of the patients were happy with the formulation of our drug and no side effects were reported. 6% of the patients reported dryness and with similar figure for burning sensation. Finally 3% experienced irritation while they have been using the product. As this reporting was identical for the left and right side we may conclude that these reported side effects could well be associated with the inactive ingredients of our formulation rather than the turmeric itself. Never the less, such level of side effects reporting only experiencing for short period at any event bearing in mind that patients with psoriasis having very sensitive skin is statistically well satisfactory (Figure 10). 

## Discussion

In recent years, plaque psoriasis is recognized as the most prevalent autoimmune disease that has a significant impact not only on the patient`s health but also on a patient’s quality of life, sometimes profoundly altering their everyday life (10). This randomized, prospective intra-individual, right–left comparative, placebo-controlled, double-blind clinical trial was aimed to investigate the topical turmeric microemulgel on plaque-type psoriasis with the view of disease management and quality of life parameters. This study was designed bearing in mind the demographic influential factors, causes and ultimately comorbidities.

There are multiple treatment options for this group of patients, including systemic therapies, topical therapies and phototherapy. These options can be used in conjunction with other drugs or on their own (11). At the present time, Cyclosporine, Calcipotriol, Retinoids, Dithranol and Coal tar are amongst the most accepted anti-psoriatic compounds. However, most of the current psoriasis topical treatments are suppressive and directed either at inducing a remission or relieving the patient’s condition (1, 12, 13). Being messy and irritations associated with Coal tar, burning and staining associated with Calcipotriol, local tissue atrophy and systemic side effects with Corticosteroids, photoreaction and exaggerated burning of Methoxsalen can be a major reason to limit their uses (1, 14, 23). 

In addition to all the mentioned therapies, topical herbal remedies have a great place in the management of psoriasis as it has lesser side effects or toxic effects in comparison to the other therapies. About 75% of the patients with mild-to-moderate psoriasis are amenable to topical treatment in their lifetime and using herbal remedies like Aloe vera, Cayenne, Chamomile, Turmeric, Flaxseed oil can create an additional therapeutic benefits (3). 

Turmeric, derived from the plant *Curcuma longa*, is a gold-colored spice commonly used in the India. Considerable interests have been focusing on curcumin which is one of the major constituent and isolated from turmeric (5, 15). The use of curcumin for the treatment of various skin diseases like scleroderma, psoriasis and skin cancer has been suggested by many scientists and clinicians around the world. Curcumin has the ability to down regulate the receptors of 5-LOX, COX-2, TNFα, IL-6, IL-8 and IL-1 cytokines in order to reduce the redness and inflammation. Curcumin decreases the expression of proinflammatory cytokines and growth factors such as interleukin 6 and IL-8 in human keratinocytes (21). Phosphorylase kinase (PK), a calmodulin containing enzyme, is expressed at significantly higher levels in patients with psoriasis. Higher levels of phosphorylase kinase (PK) activity, glycogenolysis and phosphorylation are associated with increased psoriatic activity. Curcumin is a potent, selective and non-competitive phosphorylase kinase (PK) inhibitor and was shown to reduce the PK levels in psoriatic patients (16, 21). Our data and clinical observations throughout the study have demonstrated the ability of this product to be an additional option in conjunction with other therapeutic options to treat this medical condition.

In a clinical study by Heng *et al.* (2000) it was reported that five out of ten patients had 90% resolution of psoriasis after 2-6 weeks of treatment with curcumin gel, and the rest of the patients had 50-85% improvement after 3-8 weeks of treatment. Curcumin was found to be more effective in reducing the PK levels when compared with Calcipotriol, a vitamin D3 analogue in psoriasis. The resolution of psoriasis in the curcumin-treated group was superior in comparison to the Calcipotriol or untreated group (16). Various topical formulations are investigated by researchers around the world for their absorption properties. One of the most recent studies by khiljee *et al.* (2010) has investigated the three dosage forms of turmeric extract; a microemulsion, a gel, and an ointment with the aim of evaluating and comparing their release properties from topical preparations using franz cells. After 6 hour, it was observed that the microemulsion had the fastest release through nylon membrane and cellulose membrane compared with the gel or ointment (9). Turmeric microemulgel (our patented formulation) in this study was based on the original microemulsion idea; however, improvement to the formulation was made to compensate for the shortfalls of the previous microemulsion formulations.

In patient surveys between 2001 and 2008 by the national psoriasis foundation, 33% of patients with mild and 60% of patients with moderate-to-severe disease reported that this medical condition was a significant problem in their everyday life activities. The negative effect on the psychological and social dimensions of life can be greater than those resulting from life-threatening illnesses such as myocardial infarction. Psoriasis can negatively affect patient’s lifestyle, emotional well-being, social life and ability to work (2). 

 Currently, there are many ways and questionnaires to measure the quality of life in patients with psoriasis. Psoriasis quality of life questionnaire is referred as the major well recognized method in assessing quality of life in patients with psoriasis and psoriatic arthritis (10, 17). Never the less, it is suggested that some of the therapeutic options may improve the lesions but don’t necessarily improve the quality of life and ultimately are not providing the maximum clinical benefit. In general, there is correlation between quality of life parameters and the PASI scores on individual patients. In recent studies the trials of new agents such as Alefacept, Efalizumab, and tumour necrosis factor α (TNF α) inhibitors such as Etanercept and Infliximab have demonstrated that patient`s quality of life evidently improves with treatment. This means that, the PASI scores may show statistically significant improvement early in clinical trials and therefore the analysis of quality of life parameters can be used to confirm if these changes are clinically significant or meaningful in real life case scenarios (17, 18, 19). Bearing that in mind we have investigated the similar parameters using the same method to evaluate the turmeric microemulgel. Interestingly, some pattern of improvement were observed where the PASI score was reduced when turmeric microemulgel was used in comparison to the placebo (Table 2). Another study has investigated the relationships between changes in quality of life and severity of the disease. Itching, as one of the mentioned quality of life parameters were improved when patients with chronic plaque psoriasis were receiving intermittent short courses of Cyclosporin. The study demonstrated that the short courses of Cyclosporin clearly improves the overall outcome of the therapy not only by the proposed pharmacological mode of action but also when the itching was reduced the “good feeling” effects may also play an additional contributing role in the overall management and wellbeing of the patients (20). Likewise, using the turmeric microemulgel in our study has demonstrated that the level of itching was improved during 9 weeks trial. At the baseline 35% of the patients reported to have pruritus as a problem interfering with their everyday activities. However, using turmeric microemulgel has led to a meaningful reduction in the number of patients, 11% in comparison to the beginning of the study (Figure 4). In parallel to that, factors such as being self-conscious or embarrassed about the problem or interference with daily and social activities which could be important wellbeing factors were enhanced and therefore creating a better overall response to the treatment (Table 2).

The results of this study indicate that at the baseline 76.5% of the patients announced that the disease worsened by the stress (Figure 1). By the end of our study, the reported quality of life parameters were mainly evolved and signs of good feelings were reported by the patients. The author does believe that could be directly linked to the fact that the level of stress was lessened by the use of proposed microemulgel and patients were responding to the visual appearance of the lesions (Figure 11). 

Furthermore, the mean psoriasis area and severity index elements (shown in Figures 5 to 8) such as redness, thickness, scaling and area of involvement of the plaques in treated lesions in comparison to the placebo was improved (P<0.05). This improvement was equally observed in comparison to the baseline (P<0.05), meaning that the use of turmeric microemulgel can have prominent additive effects in the management of the plaque psoriasis.

In summary, turmeric microemulgel was well tolerated with trivial number of reported side effects (Figure 10). It appeared to be effective in some degree in resolution and improvement of the investigated lesions compared to the baseline and placebo (Figure 11). Based on our findings, the author believes that the new proposed microemulgel may be considered as an alternative therapeutic topical option in some patients and in all likelihood as an add-on therapeutic option for many patients suffering with plaque psoriasis. 
